# A year of progress

**DOI:** 10.1308/003588413X13511609956976

**Published:** 2013-01

**Authors:** Colin Johnson

It has been a satisfying year for the *Annals*, building on the progress of previous years and enabling us to look forward to 2013 with confidence. We continue to receive large numbers of submissions. In 2012 over 1,000 articles were offered to us, of which a quarter were research papers. The quality of these research papers has improved and we now receive very few audits. (We rarely publish single-unit audits.)

During this year, we have managed to maintain a very short time between acceptance and publication (on average about 3–4 months), and our thanks go to an excellent production team, our copy editor Tara Nikovskis and our new typesetters Hobbs, who have done an excellent job in their first year. We said goodbye to Jon Hackett, our Production Manager, who has moved to an academic post. We are fortunate to have Matthew Whitaker, who stepped into his shoes and continued the high standard that this role requires.

We continue to rely heavily on our reviewers (who are acknowledged elsewhere in this issue). They help maintain the high standard of research publications, reflected again in our rising impact factor, which now stands at 1.231.

During 2012, we have continued to improve the functions of our online submission system. This is getting a further overhaul in the coming weeks, when authors submitting research papers should find it easier to use. We recognise that our existing submission channel including both the *Annals* and the *Bulletin* caused some confusion so we have tried to make the separate channels of submission for either the *Annals* or the *Bulletin* clearer to ensure that papers are considered appropriately from the outset.

Our Technical Section continues to flourish under the editorship of Bruce Campbell. This section is a constant source of interest, and here too we have reduced the time between acceptance and publication. A notable success this year has been collection of all the published technical notes and tips into a single volume. This has only been possible because of the hard work of the editors Mike McCarthy and Bruce Campbell as well as Adam Brownsell from the Publications Department. Over 700 articles have been compiled, sorted and made available on the College website and can be searched and downloaded free of charge, allowing readers to browse and search through the combined experience and knowledge of hundreds of surgeons gathered over a decade of practice.

Our first Associate Editor has completed her term of office this year. Jyoti Shah has made a tremendous contribution in this role and has taken responsibility for editing review articles. She has now moved into a ‘senior’ position as Review Editor. Jyoti also managed the publication of a series of reviews of perioperative care and, after a brief rest, this series will return later in the year. We now have a new Associate Editor, who will have the opportunity to learn from the inside about scientific journal publication. Masood Khan, a consultant urologist in Leicester, will be developing his own project to enhance the content of the *Annals* and *Bulletin*.

Dual publication remains a recurring issue. A further case was brought to our attention by an alert reader. (See notice on page 25). Authors should be aware that any material published in paper or online formats should be original and that dual publication represents serious publication malpractice. The *Annals* continues to support the high standards of the Committee on Publication Ethics in relation to all such matters.

In this issue we have included a notice from the National Institute for Health Research National Surgery Specialty Group. This underlines our commitment to research in all the specialties of surgery, and we will publish regular updates of surgical trials in progress in the UK and approved by this group.

Looking forward to the New Year, we will maintain our average acceptance-to-publication time below four months and look to improve manuscript handling. On behalf of the College, the *Annals* has been involved in working with the *British Journal of Surgery* to establish a one-day course entitled *How to Write a Surgical Paper*. This is based heavily on the course developed by Professor Derek Alderson for the *British Journal of Surgery*. We at the *Annals* feel it is appropriate that the College should offer this excellent training course to surgeons in all disciplines. The first course will run on 1 March and we hope it will become a regular feature of the College education programme.

Finally, we have had two successful cover image competitions with a range of interesting and varied images submitted by readers. This year we will carry a series of illustrations related to surgical operations, prepared by surgical illustrators. There will be no competition for an overall prize but readers who require the services of an illustrator will be able to find contact details attached to each contribution. We hope you enjoy these images as well as the scientific content of the *Annals* during the coming year.

